# Microbiological and biochemical findings in relation to clinical periodontal status in active smokers, non-smokers and passive smokers

**DOI:** 10.18332/tid/104492

**Published:** 2019-03-21

**Authors:** Burcu Kanmaz, Gwyneth Lamont, Gülcan Danacı, Himabindu Gogeneni, Nurcan Buduneli, David A. Scott

**Affiliations:** 1 Department of Periodontology, Faculty of Dentistry, İzmir Democracy University, İzmir, Turkey; 2 Department of Oral Immunology and Infectious Diseases, School of Dentistry, University of Louisville, Louisville, United States; 3 Department of Periodontology, School of Dentistry, Ege University, İzmir, Turkey

**Keywords:** bacteria, cytokines, environmental tobacco smoke, matrix metalloproteinases, saliva

## Abstract

**INTRODUCTION:**

Cigarette users are more susceptible than non-smokers to periodontitis, a bacterial-induced, inflammation-driven, destructive disease of the supporting tissues of the teeth. We hypothesized that clinical periodontal findings and microbiological and/or inflammatory marker levels would be intermediate in those exposed to environmental tobacco smoke compared to active smokers and non-smokers.

**METHODS:**

Sixty individuals were recruited from a University periodontal clinic and assigned as non-smokers, active smokers or passive-smokers according to their self reports. Clinical periodontal measurements, comprising plaque index, probing depth (PD), clinical attachment level (CAL) and bleeding on probing, were recorded at six sites per tooth. Cotinine levels were determined in whole saliva samples by EIA. *Treponema denticola* and *Porphyromonas gingivalis* infection was determined by PCR, while matrix metalloproteinase-8 (MMP-8) and interleukin-8 (IL-8) concentrations were determined by ELISA.

**RESULTS:**

Study groups were subsequently reassigned in accordance with the cotinine data. The smoker group exhibited higher mean PD and CAL values compared to the non-smoker group (p<0.05). Passive-smokers exhibited PD and CAL values smaller than those of the active smokers and greater than those of the non-smokers, but the differences were not statistically significant. PD and CAL values correlated with cotinine concentrations (p<0.05). *P. gingivalis* infection was noted in most subjects, irrespective of smoking status. *T. denticola* infection was noted in 4/23 (17.4%) smokers, 0/16 (0%) environmentally-exposed recruits and 2/21 (9.5%) non-smokers. Salivary MMP-8 and IL-8 levels were lower in smokers compared to both non-smokers and passive-smokers but the differences were not significant (all p>0.05).

**CONCLUSIONS:**

The present clinical periodontal findings provide further support for a negative, dose-related effect of tobacco exposure on periodontal health. The tendency for a more prevalent detection of *T. denticola* and for a suppressed inflammatory response observed in the smokers may partly explain the increased susceptibility to periodontal tissue destruction, but needs to be verified in larger scale studies.

## INTRODUCTION

Severe periodontitis leads to loss of teeth and impairs functions of dentition (e.g. mastication, speech, and facial esthetics). Interactions between microbial, immunological, environmental, and genetic risk factors, as well as age, sex, and race affect progression and severity of periodontitis^1^. Smoking is generally accepted as the major preventable risk factor in the incidence and progression of periodontal disease^2-4^. Non-smoking individuals, who are exposed to environmental tobacco smoke are also prone to oral and systemic diseases^3,5,6^. Accurate assessment of active and environmental cigarette smoking is therefore important in clinical periodontal research^4^.

Cotinine, the major metabolite of nicotine, is the most commonly employed biomarker of exposure to tobacco smoke. It can be measured in biofluids such as plasma, urine, or saliva^7^. Passive smokers usually have cotinine concentrations in saliva below 5 ng/mL, whereas heavy passive exposure can result in levels higher than or equal to 10 ng/mL. Levels between 10–100 ng/mL may result from infrequent active smoking or regular active smoking with low nicotine intake. Levels greater than 100 ng/mL are normally the result of regular active smoking^7^. Definition of passive smokers by salivary cotinine levels of 1–7 ng/mL has been accepted in other studies^6^.

Environmental tobacco smoke exposure can influence periodontal and salivary biomarkers related to inflammation^8,9^ while passive smokers have been reported to have 28% increased odds of periodontitis compared to those with no exposure^10^. Bacterial-induced cytokines, such as interleukin-8 (IL-8), drive leukocyte recruitment and promote gingival inflammation. Matrix metalloproteinase-8 (MMP-8) is a major protease responsible for the degradation of collagen, and other extracellular matrix components, in periodontitis^11^. Smoking is associated with a decrease in pro-inflammatory cytokine levels and perhaps with an increased proteolytic burden, including MMP-8, as we have recently reviewed^12^. Further, tobacco use clearly promotes microbial dysbiosis in the oral cavity^13^.

We hypothesized that exposure to environmental tobacco smoke may alter infection rates with certain periodontopathogens (*Treponema denticola* and *Porphyromonas gingivalis*) and influence salivary IL-8 and MMP-8 levels in a manner intermediate to that of active smokers and non-smokers. These possible alterations may subsequently affect the clinical periodontal findings in environmentally-exposed individuals.

## METHODS

### Study population

A total of 60 participants were recruited from the general clinic population of the School of Dentistry, Ege University, İzmir, Turkey between September 2016 and December 2016. The study was conducted in full accordance with ethical principles including the World Medical Association’s Declaration of Helsinki as revised in 2000 and approved by the Ethics Committee of the Ege University, School of Medicine with reference number 16-12.1/14. The study protocol was explained to and written informed consent was received from each individual before clinical periodontal examinations and saliva sampling. Exclusion criteria were: medical disorders such as diabetes mellitus, immunological disorders, hepatitis, medications known to affect gingival tissues, antibiotic treatment in the last 6 months, age < 18 years, and pregnancy. Smokers were those who reported smoking more than 10 cigarettes/day for more than 5 years, while non-smokers were those who reported that they had never smoked. Environmentally-exposed individuals were those who reported frequent or regular exposure to tobacco smoke at home and/or work. Medical and dental histories were obtained, and the individuals were grouped according to their self-reports, later to be confirmed biochemically.

### Clinical periodontal recordings

Dichotomous plaque index (PI) and bleeding on probing (BOP) were recorded as present or absent and percentage values for whole mouth were calculated for each individual. The probing depth (PD) and clinical attachment level (CAL) values were recorded with a Williams periodontal probe (Hu-Friedy, Chicago, IL) and mean values were calculated for all participants in each study group. All parameters were obtained at six sites per tooth (mesial-buccal, mid-buccal, distal-buccal, distal-palatal, mid-palatal, and mesial-palatal) in all teeth present except the third molars in each patient.

### Saliva sampling

Whole saliva samples were obtained simply by having the individual expectorate into sterile polypropylene tubes before clinical periodontal measurements or any periodontal intervention in the morning after an overnight fast, during which participants were requested not to drink (except water) or chew gum. For saliva sampling a modified version of the method described by Navazesh was used^14^. The samples were immediately frozen and stored at -40^o^C until the sample collection period was completed and thawed immediately before assays.

### Laboratory analyses

Chelex 100 (Bio-Rad Laboratories, Hercules, CA), polymerase chain reaction (PCR) primers (Bio-Synthesis Inc. Synthesis Inc., Lewisville, TX), PCR BioLabs Taq 2x mastermix and UltraPure distilled water (Invitrogen, Carlsbad, CA), Gifu anaerobic medium (GAM) (Nissui Pharmaceutical, Tokyo, Japan), brain heart infusion (BHI) medium (Becton Dickinson, Sparks, MD), and fetal bovine serum (Atlanta Biologicals, Lawrenceville, GA) were provided together with all the other media components (Sigma Aldrich, St. Louis, MO) for laboratory analyses. High sensitivity cotinine immunoassays (Salimetrics, State College, PA) were used for chemical validation of smoking status.

### Determination of salivary cotinine concentrations

Salivary cotinine concentration was measured using a high sensitivity cotinine immunoassay and a Bio-Tek Synergy H7, as previously reported^15^. The reported sensitivity of the assay is 0.15 ng/mL.

### Growth of periodontopathogens

*P. gingivalis* ATCC 33277 and *T. denticola* ATCC 35405 were grown to mid-to-late log phase in GAM and in new oral spirochete broth (with veal heart infusion replaced with BHI and KH_2_PO_4_ omitted)^16^, respectively, under anaerobic conditions (80% N_2_, 10% H_2_, 10% CO_2_) at 37°C in an anaerobic chamber (Coy Laboratories, Grass Lake, MI).

### Detection of pathogens

The presence or absence of bacterial DNA was determined by four PCR amplifications per Chelex 100 (25% mass/volume)-treated saliva sample, using primers documented in Table 1. Cycling parameters were as previously reported^15,17,18^. DNA extracted from cultured periodontopathogens and water served as positive and negative controls, respectively. *P. gingivalis, T. denticola*, and universal 16s amplicons were visualized using a special system (Lonza Flashgel system, Rockland, ME).

**Table 1:** Sociodemographic and clinical periodontal parameters in the study groups (values are given as mean ± SD unless otherwise noted)

*Demographic/periodontal variable*	*Active smokers (n=23 )*	*Non-smokers (n=21 )*	*Passive smokers (n=16 )*
**Age** (years)	38.09 ± 8.9	45.67 ± 11.4*	40.56 ± 11.34
**Gender** (males/females) (n)	14/9	7/14	13/3
**Missing teeth** (n)	3.78 ± 3.91	2.86 ± 2.92	2.75 ± 2.41
**PD** (mm)	2.28 ± 0.87^†^	1.77 ± 0.55	2.10 ± 0.81
**CAL** (mm)	2.50 ± 1.28^†^	1.80 ± 0.58	2.19 ± 0.95
**BOP** (%)	27.62 ± 29.12	25.39 ± 25.41	28.51 ± 19.91
**PI** (%)	51.06 ± 38.04	34.95 ± 35.95	36.31 ± 34.53

* Significantly higher than the smoker group (p<0.05).

† Significantly higher than the non-smoker group (p<0.05)

### Salivary IL-8, MMP-8 analyses

Salivary MMP-8 (Bio-Rad Laboratories, Hercules, CA), and IL-8 (Bio-Synthesis Inc. Synthesis Inc., Lewisville, TX) concentrations were determined by ELISA, according to the manufacturer’s instructions.

### Data analyses

The study size was set by literature precedent^19-21^. Statistical significance was determined using InStat v3.06 (GraphPad, San Diego, CA). Parametric or non-parametric ANOVA with Tukey or Dunn post-testing, respectively, and Fisher’s Exact Test were employed, as appropriate. Correlations between clinical and/or laboratory findings were investigated by Spearman correlation analysis. Significance was set at p<0.05.

## RESULTS

### Clinical findings

The sociodemographic data and clinical periodontal findings in the study groups are presented in Table 1. There were significant differences in the gender distribution between the study groups (p<0.05). The individuals in the smoker groups were significantly younger than the non-smoker individuals (p<0.05), and were at similar age with the environmental smoker group (p>0.05). The non-smoker group exhibited the lowest values in all clinical periodontal parameters except BOP. The mean PD and CAL values were higher in the smoker group compared to the non-smoker group (p<0.05), and were similar compared to the environmental smoker group (p>0.05). Other clinical findings were not significantly different between the study groups (p>0.05).

### Laboratory findings

The salivary cotinine concentrations, frequencies of *P. gingivalis* and *T. denticola*, and salivary IL-8 and MMP-8 concentrations are given in Table 2. Cotinine concentrations clearly distinguished the study groups with highly significant differences (p<0.01). The frequency of *P. gingivalis* was similar in the study groups (p>0.05) and *T. denticola* was more frequently detected in the smoker group but the difference was not significant (p>0.05). The lowest salivary concentrations of IL-8 and MMP-8 were detected in the smoker group but the differences were not significant (p>0.05).

**Table 2:** Microbiological and biochemical findings in the study groups (values are given as median Q1-Q3 unless otherwise noted)

*Microbiological/biochemical parameter*	*Active smokers (n=23 )*	*Non-smokers (n=21 )*	*Passive smokers (n=16 )*
**Salivary cotinine** (ng/mL) (mean ± SD) (min–max)	403.54±273.46* (19.19–944.93)	0.54±0.29 (0.00–0.99)	2.27±1.68 (1.11–6.10)
***P. gingivalis frequency*** (+/-)	20/3	18/3	13/3
***T. denticola frequency*** (+/-)	4/19	1/20	1/15
**IL-8** (ng/mL)	882.84 (442.10–1506.16)	812.60 (358.86–1138.79)	1103.13 (459.22–2255.20)
**MMP-8** (ng/mL)	72.03 (30.58–180.07)	134.89 (58.70–285.69)	159.89 (27.90–411.29)

* Significantly higher than the other groups (p<0.05).

### Correlations

Spearman correlation analysis indicated significant positive correlations between the number of missing teeth and age (p<0.05). BOP values showed significant positive correlations with PI, PD, and CAL (p<0.05). Salivary cotinine concentration was positively correlated with PD and CAL (p<0.05). Salivary MMP-8 and IL-8 concentrations were positively correlated (p<0.05). MMP-8 concentration was correlated with PD, CAL, and BOP (p<0.05). All these correlations were strongest in the active smoker group.

## DISCUSSION

The major etiological factor for periodontal diseases is the microbial dental plaque. However, this group of chronic, infectious, and inflammatory diseases has a multifactorial etiology, and environmental factors, such as tobacco smoking, play a significant role in their severity and prognosis. Detrimental effects of active smoking on the occurrence and severity of periodontal diseases are well documented. However, there are limited studies investigating the potential effects of environmental smoking on periodontal health. The present study was conducted to comparatively evaluate clinical, microbiological, and biochemical findings in active smokers, non-smokers and environmentally exposed individuals. The major limitation of the present study is the rather small number of participants.

Chemical validation of smoking status is of utmost importance in studies that focus on the possible effects of smoking. Self-reported tobacco use and exposure are commonly employed but are often unreliable^22^. Therefore, in the present study the group assignment of the participants was based on the salivary cotinine concentrations.

Clinical periodontal findings tend to deteriorate with increasing age. Accordingly, the present findings indicated significant positive correlation between increasing age and number of missing teeth. However, the individuals in the active smoking group were significantly younger than the other two groups, but exhibited worse clinical periodontal findings in terms of the visual tissue destruction parameters. Detrimental effects of smoking may explain this finding. As expected, the non-smoker group revealed significantly lower values in all clinical periodontal parameters except the percentages of BOP. This is likely explained by the well-documented suppressive effects of smoking on vascular response^12^.

Possible relationships between active smoking and exposure to environmental smoke and the prevalence of periodontal diseases have been previously examined among 1167 young Japanese women with a mean age of 31.5 years^23^. In another study from Japan, salivary levels of IL-1β, lactoferrin, albumin and aspartate aminotransferase (AST) were found to be increased in passive smokers^24^. The authors concluded that active, but not passive, smoking was associated with an increased prevalence of periodontal disease. A systematic review concluded that the association between environmental tobacco exposure and periodontal disease remains debatable and requires further investigations^3^. The present study suggests some interaction between environmental smoking and worsening of clinical periodontal findings.

In a recent large-scale study from US, environmental smoking exposure was reported to increase the risk of an individual to develop periodontitis^10^. Oral examination data from NHANES study from 2009 to 2012 were used and the participants’ sociodemographic variables were investigated, whereas environmental tobacco exposure was determined according to the serum cotinine concentrations. However, another similar study reported weak associations between environmental smoking and periodontitis in Latinos^25^. The association between exposure to environmental tobacco smoke and periodontitis endpoints were evaluated in a systematic review and meta-analysis^26^. The authors concluded that associations of PD and/or CAL were stronger with cotinine-measured exposure than self-reported exposure and emphasized the detrimental effects of environmental smoking on periodontal health. The present finding of significant positive correlations between salivary cotinine concentrations and PD/CAL values in passive smokers, provides further support for the dose-response effect of smoking on periodontal disease, at least within a population resident in Turkey.

While the salivary burden of IL-8 and MMP-8 were increased in passive smokers, relative to the other smoking groups, these differences failed to reach the level of statistical significance. The lack of significant difference can be explained by the wide range of values obtained in the participants reflected in high standard deviations. On the other hand, prevalence of periodontopathogens is more likely to show a significant association with levels of inflammatory markers in gingival crevicular fluid samples rather than with salivary content, as periodontitis is a highly site-specific disease. Accordingly, numerous significant correlations have been reported between GCF cytokine levels and *P. gingivalis, F. nucleatum*, and *P. intermedia T. denticola* levels at sites of clinically healthy periodontium and also at sites with clinical diagnosis of periodontitis^27^. Further studies with larger populations may reveal significant relationship between salivary and/or gingival crevicular fluid biomarkers and environmental exposure to cigarette smoke.

## CONCLUSIONS

Within the limits of the present study, it can be concluded that environmental smoking may have detrimental effects on periodontal health. Furthermore, the data generated provide a robust basis for power calculations required to facilitate future, larger studies that will better clarify the interaction between environmental smoking, key disease biomarkers, and periodontal diseases.

## CONFLICTS OF INTEREST

Authors have completed and submitted the ICMJE Form for Disclosure of Potential Conflicts of Interest and none was reported.
